# Letter to the Editor about “Fresh human neck dissection specimens as vascular models for head and neck microsurgery training”

**DOI:** 10.1097/JS9.0000000000004553

**Published:** 2025-12-11

**Authors:** Sergey Minaev

**Affiliations:** Head of Department of Pediatric Surgery, Stavropol State Medical University, Stavropol, Russian Federation

*Dear Editor,*

I read with great interest the article Zheming Zhang *et al* “Fresh human neck dissection specimens as vascular models for head and neck microsurgery training”^[1]^, which proposes an innovative vascular training model created from fresh human neck dissection specimens. The authors contribute to an essential and evolving area of surgical education. However, after critically analyzing the methods, results, and interpretations, I would like to offer several constructive comments to strengthen the scientific validity and future development of this line of research.

The primary limitation of the study lies in its comparison strategy. The authors validate their human vascular model exclusively against chicken wing vessels (which are unfortunately not accurate enough and anatomically mismatched). Validating high-fidelity trainers requires models that replicate human vascular properties, such as perfused cadaveric tissues, human placental vasculature, or biomimetic 3D-printed phantoms^[2]^. Comparing a human-derived model only with chicken tissue may inflate perceived realism while not fully demonstrating its superiority over more advanced simulators currently used in microsurgical curricula.

Another important issue concerns the study’s methodological and statistical structure. Although the authors report feedback from trainees and senior surgeons, assessed using the MiSSES instrument, the evaluative framework remains incomplete. The study does not outline how the sample size was determined, nor does it present any power calculation, measures of effect magnitude, agreement between evaluators, or safeguards against type I error when multiple comparisons are made. Current approaches in surgical training place much greater weight on measurable performance outcomes than on subjective impressions. Validating a model today typically involves objective tools, such as OSATS, the Global Rating Scale, or direct patency testing – rather than relying solely on how participants feel about the exercise. In the absence of these quantitative benchmarks, it is challenging to determine whether the model genuinely improves microsurgical skill or merely elicits favorable subjective responses.

Equally concerning is the reliance on a preservation technique −30% glycerol at −20°C that is neither well established nor supported by vascular preservation guidelines. Recent laboratory work on tissue preservation shows that blood vessels maintain their endothelial structure and mechanical properties only when stored in cryoprotective solutions containing DMSO and held at extremely low temperatures – usually close to −80°C or even lower^[3]^. In this context, the changes observed by the authors – namely, enlargement of the vessel lumen and thinning of the wall after several weeks of storage – stand in clear contrast to their overall positive evaluation of the preservation method. Taken together, these observations point to the need for a preservation approach explicitly developed for vascular tissue, rather than one adapted from studies of unrelated biological materials.

In addition, although the authors refer to artificial intelligence and the TITAN reporting framework^[4]^, the study itself does not make use of any AI-based assessment tools. If the intention was to align the study with current methodological trends, the authors could have strengthened the work by applying computer vision tools to quantify technical performance – for example, by automating OSATS-based scoring, assessing suture placement and spacing, or analyzing vessel geometry through segmentation algorithms. Had the authors actually integrated any AI-based tools into their evaluation, the reference to AI guidelines would have seemed warranted and more in step with how surgical simulation research is developing today. Without such practical use, the mention of AI comes across more as an editorial flourish than as a substantive element of the study^[5]^.

Overall, the authors offer a creative and easily obtainable approach for practicing microsurgical techniques using tissue from neck dissections. Yet, several important shortcomings – particularly in the study design, the way safety issues are addressed, and the absence of robust objective performance metrics – limit how well this model can function in routine training settings. These core issues must be resolved before the work can make a significant contribution to surgical simulation.

Thank you for considering these remarks for publication in the *International Journal of Surgery*. They may stimulate constructive discussion and help refine future research in this important field.

## Data Availability

Not applicable.

## Research registration unique identifying number (UIN)

There are no conflicts of interest.
